# How to Prescribe

**Published:** 1897-06

**Authors:** 


### How to Prescribe.

The following good advice on prescribing was given by Dr. J. T. Graham to the medical men of Virginia at the last annual meeting of the State Association: "Now, before giving a drug, the physician, after being sure of his diagnosis, should have a clear conception of the effects the drug he is going to prescribe will have on the functions of the body—that is, he must know its physiological action; and then, beginning with a small dose, give the selected drug until its known physiological effect is produced—bearing in mind that the dose given in our books on materia medica is the one to be started with. So, then, when the diagnosis is made, and the drug selected, give it full time to produce its effect before changing to some other remedy."

